# Diagnostic accuracy of oximetry for obstructive sleep apnea: a study on older adults in a home setting

**DOI:** 10.6061/clinics/2021/e3056

**Published:** 2021-09-20

**Authors:** João Carlos Fraga da Rosa, Alessandra Peres, Luciano Gasperin, Denis Martinez, Vania Fontanella

**Affiliations:** I Universidade Federal de Ciencias da Saude de Porto Alegre, Porto Alegre, RS, BR.; II Universidade Federal do Rio Grande do Sul, Porto Alegre, RS, BR.

**Keywords:** Obstructive Sleep Apnea, Polysomnography, Oximetry, Sensitivity and Specificity

## Abstract

**OBJECTIVES:**

Owing to the fact that obstructive sleep apnea (OSA) is an underreported disease, the strategy used for the diagnosis of OSA has been extensively dissected to devise a simplified process that can be accessed by the public health services. Polysomnography (PSG) type I, the gold standard for the diagnosis of OSA, is expensive and difficult to access by low-income populations. In this study, we aimed to verify the accuracy of the oxyhemoglobin desaturation index (ODI) in comparison to the apnea-hypopnea index (AHI) using a portable monitor.

**METHODS:**

We evaluated 94 type III PSG home test results of 65 elderly patients (69.21±6.94 years old), along with information, such as the body mass index (BMI) and sex, using data obtained from a clinical trial database.

**RESULTS:**

A significant linear positive correlation (r=0.93, *p*<0.05) was observed between ODI and AHI, without any interference from sex, BMI, and positional component. The sensitivity of ODI compared to that of AHI increased with an increase in the severity of OSA, while the specificity of ODI in comparison to that of AHI was high for all degrees of severity. The accuracy of ODI was 80.7% for distinguishing between patients with mild and moderate apnea and 84.4% for distinguishing between patients with moderate and severe apnea.

**CONCLUSION:**

The ODI values obtained in uncontrolled conditions exhibited high sensitivity for identifying severe apnea compared to the AHI values, and correctly identified the severity of OSA in more than 80% of the cases. Thus, oximetry is promising strategy for diagnosing OSA.

## INTRODUCTION

Obstructive sleep apnea (OSA) is a global public health problem associated with serious clinical outcomes, such as cardiovascular diseases (1). It is estimated that at least 730 million individuals aged 30 to 69 years are affected by OSA, with a higher prevalence in China, the United States, Brazil, and India (2). Studies indicate that senescence leads to an increase in the prevalence of OSA, a condition characterized by a decrease in muscle activity in the upper airway, pharyngeal dilator reflex, and lung capacity, and a higher prevalence of comorbidities (3,4).

Polysomnography (PSG) type I is the gold standard for the diagnosis of OSA (5). This test is performed in a specialized sleep laboratory, where an experienced team monitors and interprets the results. Owing to its high cost, a majority of the population cannot access PSG type I. As the PSG type I-related protocols mandate that the patient sleep in the laboratory, the reliability of the test might be compromised, especially increased latency for rapid eye movement (REM) sleep might be observed as the laboratory environment is sufficiently different from the home environment. However, portable monitors (PM) for PSG type III can be used at home (5) at a much more affordable cost (6). In addition, other confounding factors exist in the diagnosis of OSA; these include the positional component, body mass index (BMI), sex, age, and associated comorbidities (7-9).

Thus, it is necessary to investigate simpler and less expensive methods to expand access to OSA diagnosis. Previous studies (10-21) have assessed the ability of pulse oximetry to diagnose OSA; they observed that compared to the apnea-hypopnea index (AHI), the oxyhemoglobin desaturation index (ODI)—measured using the oximeter—exhibited high sensitivity and specificity for diagnosing OSA. However, in most studies, validation was performed based on comparison of the test results in a controlled environment—*e.g.*, PSG type I in the laboratory—with different sampling designs, thus questions are raised regarding the diagnostic viability of ODI values measured in a laboratory environment for extrapolation in an unsupervised home environment.

This study assessed the accuracy of ODI for diagnosing OSA—in comparison to that of the gold standard, *i.e.*, AHI obtained through PSG type III—at home and without technical intervention, in elderly individuals with clinical complaints compatible with a diagnosis of OSA.

## METHODS

This was a randomized controlled trial (RCT) that was performed in a blinded manner; this cross-sectional, multicentric study was approved by the National Commission for Research Ethics under report N. 2,581/776. This study employed a database of patients selected for the RCT, which aimed to evaluate the response to OSA treatment using intraoral mandibular advancement appliances, conducted between 2018 and 2020, containing 116 consecutive tests of type III PSG, with concomitant ODI and AHI records using the same equipment (ApneaLink™, ResMed Corporation, Poway, CA, USA). The participants were previously instructed on the use of the device and placement of sensors (nasal cannula, pulse oximeter, and respiratory effort belt) for later unsupervised use at home.

Patients with associated comorbidities (pulmonary, neurological, and psychiatric diseases) and under 60 years of age were excluded. In addition, patients who did not satisfactorily exhibit the respiratory flow in the cannula, had no oximetry registration, or those with a recording time shorter than 4 h were excluded from the present analyses. The final sample consisted of 94 examinations from 65 patients, 45 of whom underwent only one examination. All other patients underwent more than one examination for treatment follow-up. 

The PSG results were retrospectively evaluated by a trained and calibrated technician without access to clinical data, as per the criteria recommended by the American Academy of Sleep Medicine (AASM) (5). The criterion for oxyhemoglobin desaturation was a 3% decrease in the baseline automatically obtained by the equipment. The degree of disease severity was defined according to the following parameters: normal, AHI<5 events/hour; mild, 5≤AHI<15 events/h; moderate,15≤AHI<30 events/h; and severe, AHI≥30 events/h (5).

The results of examinations using the equipment—and subsequently interpreted—were tabulated, and the total AHI and ODI values listed according to the patient’s position during registration. OSA was considered to have an associated positional component when, in the non-supine position, the AHI was reduced by at least 50% of the total AHI (22). BMI and sex variables were obtained from medical records of the patients. The individuals were categorized as normal weight (BMI≤24.9), overweight (≥25.0 and ≤29.9), and obese (≥30) (23).

Statistical analysis was performed using R (version 3.5.1; The R-project for statistical computing). This included descriptive analyses of the variables of interest, correlation between variables (Pearson correlation coefficient), creation of a Bland-Altman plot, and calculation of the sensitivity, specificity, accuracy, area under the receiver operating characteristics (ROC) curve, and the Youden index.

## RESULTS

The final sample included 94 test results from 65 patients, with a mean age of 69.21±6.94 years, with 63.07% females with a mean BMI of 30.13±5.19. With respect to the classification of the degree of severity of OSA, 7 patients (7.54%) exhibited normal results in the examinations, 27 (28.72%) had mild grade OSA, 31 (32.98%) had moderate grade OSA, and 29 (30.85%) had severe grade OSA. Sleep parameters are listed in Table 1.

**Figure 1:**
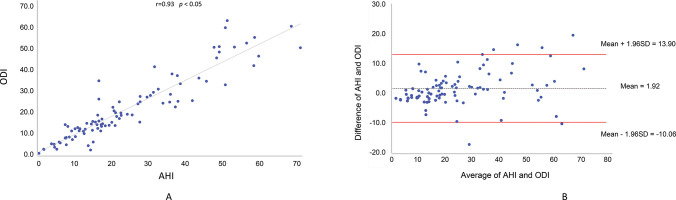
Correlation between the oxyhemoglobin desaturation index (ODI) and the apnea-hypopnea index (AHI) in the entire sample (a). Bland-Altman plot showing the comparison between AHI and ODI with confidence interval of 95% (b).

A significant linear and positive correlation (r=0.93, *p*<0.05; Pearson correlation coefficient) was observed between the ODI and AHI, with 86.17% of the variability of AHI explained by the ODI (Figure 1A). Figure 1B shows the Bland-Altman plot of the agreement between the two variables.

Pearson correlation coefficient was used to verify if the correlation between AHI and ODI was subject to any interference from sex, BMI, and the positional component (Table 2). Sex and BMI did not significantly interfere with the correlation between ODI and AHI (*p*<0.05). However, the correlation between ODI and AHI was found to be null when evaluated as a function of the positional component. 

ODI overestimated the severity of OSA in 7 patients, of whom 3 had mild apnea, 3 had moderate apnea, and 1 had severe apnea. Conversely, it underestimated OSA severity in 8 patients with mild apnea and in 8 patients with moderate apnea. Table 3 shows that the sensitivity of ODI is increased, and the specificity is maintained even upon increased OSA severity. ODI could distinguish between patients with mild or no apnea and patients with moderate or severe apnea (AHI>15 cut-off) with a sensitivity and specificity of 80% and 94%, respectively (Table 3).

**Figure 2:**
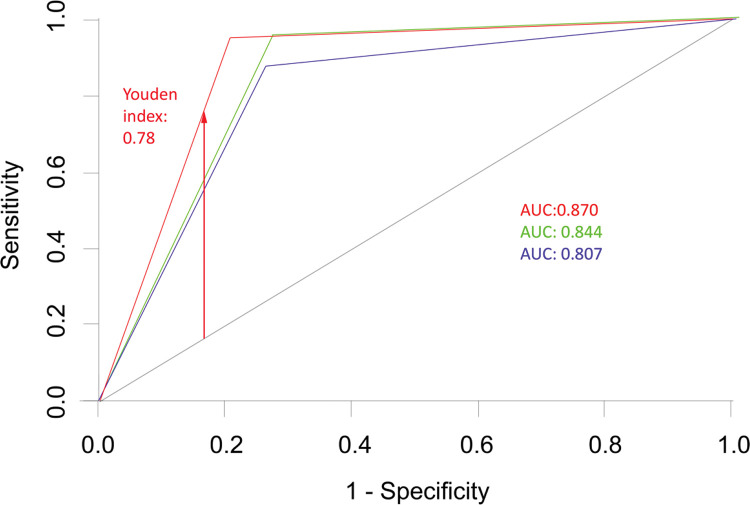
ROC curves expressing the ability of ODI to distinguish between patients with mild and moderate apnea (curve 1 - blue), between patients with moderate and severe apnea (curve 2 - green), and between patients with mild or no apnea and patients with moderate and severe apnea (AHI>15 cut-off, curve 3 - red). ROC: receiver operating characteristics; AUC: area under the curve; AHI: apnea-hypopnea index; ODI: oxyhemoglobin desaturation index.

The accuracy of ODI—as estimated using the area under the ROC curve—was 0.807—for distinguishing between patients with mild and moderate apnea—and 0.844 for distinguishing between patients with moderate and severe apnea. When an AHI ≥15 was considered as cut-off, the accuracy was 0.870, with a Youden index of 0.78 (Figure 2).

## DISCUSSION

The main results of this study were the significant linear and positive correlation between ODI and AHI for OSA diagnosis in elderly individuals—without interference from sex, BMI, and positional component—with increased sensitivity and specificity for identifying increased disease severity and the potent capability to distinguish between patients with mild and moderate apnea and patients with moderate and severe apnea.

Most studies involving validation of the use of oximetry as a screening method for OSA involve evaluation in a controlled environment and using the PSG type I test as reference, allowing immediate resolution of the changes in signal quality. Despite achieving favorable results, some of these studies evidenced the need to extrapolate the methodology to the uncontrolled home environment (10,16). Thus, this study aimed to fill this gap by analyzing ODI and AHI simultaneously obtained by the same PM.

In an uncontrolled environment, there is great variability in signal quality, which can lead to false positives and negatives (19,24). In the present study, the variability in signal quality was controlled at the time of test result interpretation. The finding that a significant number of examinations (n=22, 25.5%) presented insufficient or inaccurate nasal flow signal, is a strong indicator of the greater potential of oximetry as a parameter in home examinations. Home examination, even in the presence of a nasal cannula, oximeter, and respiratory effort belt, makes sleep more compatible with the actual routine of the patient, as it does not require adaptation to the environment. In this sense, by reducing the number of peripherals used in the test, it is possible to obtain results that are comparable to a night's sleep, without significant interference.

As OSA is a disease with a high prevalence (1,25) and is still largely unknown to the general population, it is assumed that simpler diagnostic methods, which can be offered to a large part of the population, can provide advantageous results (7,9). In Brazil, the rate of OSA underdiagnosis is high and that the Brazilian public unified health system has diagnosed OSA in only 0.07% of the population (25).

Two RCTs (6,17) indicate that PSG type III obtained by PM is sufficient and less expensive for OSA diagnosis and, and that the results of the test enable decision making regarding the clinical treatment of OSA. However, few specific studies have investigated OSA diagnosis in the elderly (26,27), who are expected to have greater difficulty in adapting to the peripherals used in PSG and a higher rate of comorbidities (24). As a differential of this study, the in the sample in the present study included elderly individuals (≥60 years of age). 

The use of oximetry has been extensively investigated in the last decade as an auxiliary method in OSA diagnosis by expanding access to diagnosis, thereby improving the quality of life of more vulnerable populations (24,25). The results of this study, as well as the existing literature (7-16,19,21,25-30), indicate that, greater the degree of apnea, greater the chances of correct diagnosis based on ODI. A recent systematic review (7) demonstrates the methodological heterogeneity with respect to the following: criterion for ODI (3% and 4%), cut-off point for the classification of the degree of apnea, and the different statistical methods used.

Assessment of the sensitivity of ODI compared to that of AHI in patients with different degrees of apnea severity revealed results similar to those presented in the literature for severe apnea in the same age group (26-27). One such study (27) evaluated the parameters of 2, 4, and 6% oxyhemoglobin desaturation from the baseline for event recording, in which the 2% criterion showed better results. Recent studies (14,15,20,29) have reported the sensitivity of ODI for OSA detection to range from 42.03 to 95.1%. In this sample, sensitivity ranged from 66.7 to 95.5% and specificity ranged from 85.3 to 91.8%. The high specificity confirmed the viability of OSA diagnosis using ODI. Other studies have identified similar values for specificity, but reported the sensitivities of 42.0% (14) and 80.0% (15) for detecting mild apnea using ODI (compared to PSG type I). For moderate apnea, we found the same sensitivity as that for mild apnea (66.7%); however, specificity was slightly lower (85.3%) and in an intermediate range compared to the reports in the literature (69.1 to 95.1%) (14,15,20,26,29). For severe apnea, ODI sensitivity was 95.5% and specificity was 88.9%, similar to previously reported studies (14,15,20,29).

Evaluation of the degree of correlation to explain the variability of ODI in relation to that of AHI revealed an r of 0.93 and R^2^ of 86.17% (*p*<0.05). Lin et al. (15) reported a similar result (r=0.956, R^2^=0.914, and *p*=0.001).

The variables associated with the severity of OSA (19), such as BMI, age, sex, and positional component, are commonly mentioned in the studies, yet they are not parameterized (16-18). Fabius et al. (24) mentioned age and BMI as covariables that influence the relationship between AHI and ODI, owing to greater desaturation in the elderly. In this study, this relationship was not influenced by any of these variables, as a significant correlation existed between ODI and AHI in both sexes (r=0.93) and in the different BMI categories (normal: r=0.98; overweight and obesity: r=0.87).

Patient position during examination—either supine or not—is a component that can influence the variability of AHI, and consequently, the analysis of ODI by oximetry as a diagnostic standard (31). In this study, 56% of the examinations (n=53) presented variation in the severity of OSA caused by spontaneous alternation between supine and non-supine positions, with a mean AHI in the supine position of 28.8 events/h and 16.6 events/h in the non-supine position. However, the correlation between ODI and AHI was found to be null when evaluated as a function of the positional component (r=0.02, R^2^=0.56, and r=0.11, R^2^=1.16). Although the mechanism of the positional component is not completely elucidated as a confounding factor for the diagnosis of OSA, its influence can be explained by the increase in the retropalatal space when making a shift from a supine to non-supine position (32).

Another important question addressed in the present study is the accuracy of ODI for adequately distinguishing the degree of severity of OSA, *i.e.,* 80.7% for mild *versus* moderate apnea and 84.4% for moderate *versus* severe apnea. Excellent accuracy has been reported for distinguishing between moderate and severe apnea, yet the classification methods are very heterogeneous (16,19,28,33).

This study was interrupted because of the COVID-19 pandemic, and the sample size was limited. In addition, the narrow age range can lead to confounding bias, yet it is relevant to understand the behavior of the investigated variables in the elderly. The inclusion of more than one examination of the same patient is also a limitation of validation studies. The second examination was performed to evaluate the results of the RCT. In half of the patients, improvement was observed, with a reduction in the degree of disease severity. In the other patients, no change or worsening of disease severity was observed after the intervention. It should be highlighted that the intervention resulted in symmetric variations in the two parameters evaluated (ODI and AHI), which indicated the same classification of the degree of apnea severity.

Oximetry has several monitoring indications, such as pulmonary and neurological diseases, as well as pre- and postoperative evaluations (30). Recently, the COVID-19 pandemic has resulted in the extensive dissemination of this resource (34). Advances in technology allow the results of oximetry to be recorded in real time on smartphones, thereby allowing repetitions and storage of data for several nights of sleep, which permits comparisons. These novel advancements were validated against PSG type I, and found to exhibit high sensitivity and specificity (20). A recent historical study that analyzed the use of AHI indicated the need to identify new clinical markers for diagnosing OSA (21).

## CONCLUSION

The ODI values, obtained in uncontrolled conditions, *i.e.*, at home, exhibited high sensitivity (for OSA detection) and correlation with AHI, but were limited to cases of moderate and severe apnea, without interference from sex and BMI. In addition, the technique correctly distinguished the severity of OSA in more than 80% of the patients. Thus, oximetry can contribute to the early diagnosis of OSA, especially in public services where access to PSG is limited.

## AUTHOR CONTRIBUTIONS

Rosa JCF and Fontanella V substantially contributed to the conception, design, acquisition of data, analysis, and interpretation of data, have been involved in drafting the manuscript and revising it critically for important intellectual content, have given approval for the publication of the final version of the manuscript, take responsibility for the content, and agree to be accountable for all aspects of the paper. Peres A and Martinez D substantially contributed to the conception and critical revision of the manuscript for important intellectual content, have given approval for the publication of the final version of the manuscript, take responsibility for the content, and agree to be accountable for all aspects of the paper. Gasperin Júnior L substantially contributed to the acquisition of data, has been involved in drafting the manuscript, has given approval for the publication of the final version of the manuscript, takes responsibility for the content, and agrees to be accountable for all aspects of the paper.

## Figures and Tables

**Table 1:** Sample characteristics with respect to sleep parameters.

Sleep parameters	Mean	Standard deviation	Maximum	Minimum
Recording time (h)	7:36	1.36	10:05	4:12
AHI	23.8	16.40	69.6	0.6
AHI supine position	28.8	19.46	75.3	0
AHI non-supine position	16.6	20.82	109.1	0
ODI	21.9	14.49	60.9	2.2
Minimum oxygen saturation	79.62	7.8	92	61

AHI: apnea-hypopnea index. ODI: oxyhemoglobin desaturation index.

**Table 2:** Correlation between oxyhemoglobin desaturation index (ODI) and apnea-hypopnea index (AHI) according to sex, body mass index (BMI), and the positional component.

Variables	r	R^2^
Sex		
Female	0.93	87.04
Male	0.93	84.34
BMI		
Normal	0.98	95.75
Overweight	0.87	74.73
Obesity	0.87	74.73
Positional component		
No	0.02	0.56
Yes	0.11	1.16

r: Pearson Correlation Coefficient. R^2^: Determination Coefficient.

**Table 3:** Sensitivity and specificity values of oxyhemoglobin desaturation index (ODI) to distinguish between patients with various degrees of apnea severity, *i.e.*, patients with mild or no apnea and patients with moderate or severe apnea.

Statistical test	Degree of apnea severity				Cut-off
	AHI <5	AHI 5<15	AHI 15 <30	AHI ≥30	AHI >15
Sensitivity	0.667	0.667	0.667	0.955	0.800
Specificity	0.965	0.918	0.853	0.889	0.940
